# Perioperative Treatment in Gastric Cancer: A Fast-Changing Field

**DOI:** 10.3390/cancers16234036

**Published:** 2024-12-01

**Authors:** Mafalda Costa, Catarina Lopes Fernandes, Helena Magalhães

**Affiliations:** Medical Oncology Department, Pedro Hispano Hospital, 4464-513 Matosinhos, Portugal; helena.menezesmagalhaes@ulsm.min-saude.pt

**Keywords:** gastric cancer, perioperative treatment, targeted therapies, immunotherapy

## Abstract

Gastric cancer is the fifth most common cancer worldwide and its incidence is rising. Surgery is the only curative strategy and its association with perioperative chemotherapy is now standard treatment for most resectable tumors. Despite treatment advances, disease relapse is high, even in early stages, and continued improvement in curative treatment is imperative. With deeper knowledge of gastric cancer heterogeneity, molecular subtypes, and the tumor immune microenvironment, new standard treatment strategies may emerge in the near future. This paper provides a comprehensive review of the current treatment landscape in resectable gastric cancer and future perspectives for the next decade regarding new agents such as targeted therapies, immunotherapy, antibody–drug conjugates, and the combination of multiple treatment modalities.

## Simple Summary

Gastric cancer is a common cancer worldwide, with a growing incidence. The standard treatment for early-stage and resectable tumors involves surgery combined with perioperative chemotherapy. High relapse rates significantly compromise prognosis, highlighting the need for new strategies in the perioperative setting. This paper offers a thorough review of the latest evidence regarding perioperative treatment for gastric cancer. It integrates new agents such as targeted therapies, immunotherapy, antibody–drug conjugates, and multimodal strategies, and presents future perspectives in this field.

## 1. Introduction

Gastric cancer is the fifth most common and fifth most lethal cancer worldwide, with almost one million new cases in 2022, mainly due to its high incidence in Asian countries [1]. Notably, its incidence is predicted to increase by 66% in the next 20 years, not only in older age groups but also in younger ages (<50 years old) [2].

Gastric cancer is a heterogeneous and complex disease, and an in-depth characterization is essential for a correct understanding and management of each case. Topographically, gastric cancer can be divided into cardia and non-cardia cancer, with the latter being the most common, accounting for 82% of cases. While smoking, alcohol consumption, a high-salt diet, and family history are risk factors common to both cancer locations, cardia cancer is more associated with obesity and gastroesophageal reflux and non-cardia cancer is mainly associated with Helicobacter pylori infection [3]. Not surprisingly, while non-cardia cancer seems to be decreasing, probably due to improved Helicobacter pylori infection screening and treatment strategies, cardia cancer cases are experiencing a worrying increase, including in younger patients [3].

Histologically, 90% of gastric cancers are adenocarcinomas, and they can be further subclassified into various histological subtypes, according to the World Health Organization’s (WHO) 2019 classification of digestive tumors [4], which are classically divided into tubular or Lauren intestinal type and poorly cohesive or Lauren diffuse type adenocarcinomas. Tubular adenocarcinoma has the highest incidence, is more common in men and older patients, and is associated with a better overall prognosis, while poorly cohesive adenocarcinoma is more common in younger patients, is associated with a worse prognosis, and is experiencing a progressively rising incidence [5,6].

At the molecular level, significant progress has been made in the last two decades, leading to a broader understanding of the molecular behavior of gastric cancer and allowing for a more comprehensive classification of the disease. The Cancer Genome Atlas (TCGA) project proposed, in 2014, a molecular classification of gastric cancer into four groups: genomically stable tumors (GS), chromosomally unstable tumors (CIN), tumors with microsatellite instability (MSI), and Epstein–Barr-infected tumors (EBV+) [7]. CIN was the most common subtype and was found to be associated with a tubular histology, p53 mutations, and RTK-RAS activation. MSI was the second most common subtype, associated with a high tumor mutational burden (TMB). GS tumors were found to be related to a poorly cohesive histology and higher rates of CDH1 mutations and CLDN18-ARHGAP fusions. Finally, EBV+ tumors, the least common subtype, were associated with PIK3CA mutations, PD-L1/2 overexpression, and immune cell signaling [7].

In 2015, an alternative molecular classification was proposed by the Asian Cancer Research Group, and it included mesenchymal-like tumors, MSI tumors, microsatellite-stable TP53-active (MSS/p53+) and microsatellite-stable TP53-inactive (MSS/p53−) tumors. Mesenchymal-like tumors are related to a poorly cohesive histology, younger age, high recurrence rate, and the worst overall prognosis. MSI tumors are related to a tubular histology and a high mutational burden, occur mainly in the gastric antrum, and have the best overall prognosis with the lowest recurrence rates. The remaining two subtypes predict an intermediate recurrence rate, with p53+ being associated with a better prognosis than p53- [8]. Regarding these molecular classifications, the only subgroup with therapeutic implications are MSI-high (MSI-H) tumors. These tumors have proved to be less responsive to chemotherapy and to be particularly sensitive to immunotherapy; they are the first cluster of gastric cancer patients to show benefit with immunotherapy [9].

Beyond molecular classification, some other molecular biomarkers, such as human epidermal growth factor-2 (HER-2), fibroblast growth factor receptor (FGFR), Claudin 18.2, and programmed death ligand 1 (PD-L1), have revolutionized the gastric cancer landscape, with proven prognostic and targetable value.

At the present moment, gastric surgery with D2 lymphadenectomy remains the only curative treatment for locoregional resectable gastric cancer. Considering the high rates of recurrence after surgery, complementary approaches have been tested and the current standard treatment of locoregional gastric cancer consists in perioperative chemotherapy with the triplet FLOT (5-fluorouracil, leucovorin, oxaliplatin, and docetaxel) interposed with gastric surgery [10]. Despite the improved pathological response, lower relapse rates, and prolonged overall survival achieved with this strategy [10], there is still a long way to go in order to further improve outcomes and decrease relapses in locally advanced gastric cancer. Therefore, several alternative and complementary strategies are being pursued, encompassing both validated and emerging biomarkers in gastric cancer.

This review provides a comprehensive summary of recent advances and ongoing trials on perioperative gastric cancer management and outlines future directions and perspectives in this area.

## 2. Current Practice in Gastric Cancer Perioperative Treatment

The standard of care for locally advanced gastric cancer in Europe has been based on perioperative chemotherapy since the results of the MAGIC trial and later the FNLCC ACCORD 07/FFCD 9703 trial and FLOT4-AIO were published [11,12], as summarized in Table 1.

The phase III MAGIC trial included 503 patients with esophagogastric adenocarcinoma randomized to receive perioperative chemotherapy versus surgery only. The perioperative chemotherapy regimen chosen consisted of three preoperative and three postoperative 21-day cycles of epirubicin, cisplatin, and 5-fluorouracil (ECF). The results revealed a superior 5-year survival rate with the perioperative strategy (36% vs. 23%) with a hazard ratio (HR) of 0.75 (95 CI, 0.60 to 0.93; *p* 0.009) [11].

The phase III FNLCC ACCORD07/FFCD9703 trial included mostly adenocarcinomas of the lower esophagus and the gastroesophageal junction and only 25% of gastric adenocarcinomas. In this trial, patients were randomized to perioperative chemotherapy with two or three preoperative cycles followed by three or four postoperative cycles of cisplatin and fluorouracil. The perioperative strategy had better resection rates (84% vs. 73%, *p* 0.04), and also improved overall survival, with a 5-year overall survival (OS) rate of 38% vs. 24% and a HR of 0.69 (95% CI, 0.50 to 0.95; *p* 0.02) [12].

It was only when the results of the FLOT4-AIO trial were presented in 2017 that the perioperative strategy gained strength; the trial showed even superior results with a new triplet chemotherapy regimen consisting of fluorouracil, leucovorin, oxaliplatin, and docetaxel (FLOT). The new perioperative strategy included four preoperative and four postoperative cycles with the FLOT regimen and was compared to the standard at the time, which was three preoperative cycles and three postoperative cycles of ECF or ECX (epirubicin, cisplatin, and capecitabine), as in the MAGIC trial. The trial included locally advanced esophagogastric junction and gastric adenocarcinomas (≥cT2 and/or cN+), and the final results showed a significant improvement in overall survival, with a median OS of 50 months with FLOT versus 35 months with ECX/ECF and a HR of 0.77 (95 CI; 0.63 to 0.94; *p* 0.012). There were no major concerns related to serious adverse events or toxic deaths [10].

Based on the results of the FLOT4 trial, perioperative chemotherapy with FLOT was adopted as the standard strategy for fit patients with locally advanced resectable gastric cancer. Table 1 summarizes the currently approved perioperative treatments for gastric cancer.

Future perioperative strategies for gastric cancer, integrating immune checkpoint blockade, targeted therapies, and multimodal strategies, as well as potential predictive biomarkers for response to treatment in different gastric cancer subtypes, will be further explored in this review.

## 3. New Perspectives for Gastric Cancer in the Perioperative Setting

### 3.1. Immune Checkpoint Blockade

Immune checkpoint inhibitors (ICIs) in combination with chemotherapy showed promising results in advanced, PD-L1-positive esophagogastric junction and gastric cancers (GEJ/GCs) [13,14,15]. These results led to the design of a number of trials with ICIs in the perioperative setting for GEJ/GC.

Three large randomized phase III trials—AIO DANTE, MATTERHORN, and KEYNOTE-585—were designed to evaluate the benefit of adding ICIs to perioperative chemotherapy, as summarized in Table 2.

KEYNOTE-585 is the phase III trial presenting more mature data in terms of event-free survival (EFS) and pathological complete response (pCR). In this trial, patients receiving perioperative chemotherapy, with a backbone of cisplatin plus capecitabine (XP) or 5-fluorouracil (FP) in the first cohort and FLOT in a second smaller cohort, were randomly assigned to receive a combination with pembrolizumab (anti-PD1) followed by adjuvant pembrolizumab versus placebo for up to eleven cycles. Regarding pCR, there was an improvement in the chemoimmunotherapy arm, with a Δ of 11.4% in both the main cohort and main plus FLOT cohort analysis. EFS was superior with chemoimmunotherapy (44.4 vs. 25.3 months), and although it was maintained in long-term follow-up, these differences were not statistically significant (HR 0.81, *p* 0.0198), mainly due to the complex statistical analysis of the trial. At the final analysis of KEYNOTE-585, median overall survival (mOS) was 71.8 vs. 55.7 months with perioperative pembrolizumab plus chemotherapy vs. placebo plus chemotherapy (HR 0.86; 95% CI, 0.71–1.06). Subgroup analysis for EFS and OS revealed a higher benefit in MSI-H patients and in tumors with PD-L1 expression by combined positive score (CPS) > 10, which may be clinically relevant to future trials. One of the limitations of KEYNOTE-585 was the chemotherapy backbone, since it was designed before FLOT became the standard of care. Only 20% of patients received perioperative chemotherapy with the current standard of FLOT and so the majority of patients were treated with an inferior chemotherapy regimen, which may have contributed to these results [16].

Further studies are ongoing and may overcome some of these limitations, such as the chemotherapy backbone and the selection of biomarkers for ICI benefit. In the phase III MATTERHORN trial, all patients received perioperative chemotherapy with the FLOT regimen combined or not with durvalumab (anti-PDL1) followed by durvalumab for up to 10 cycles. At the pre-planned interim analysis, the addition of durvalumab to perioperative FLOT demonstrated a statistically significant and clinically relevant improvement in pCR and near-pCR, with an absolute between-arm difference in pCR and near-pCR rate of 12% and an improvement in downstaging, with more participants achieving T0 and N0 (ypT0, 21% vs. 10%; ypN0, 47% vs. 33%). A total of 63% of patients in each arm completed the four postoperative doses of FLOT. Also, in the subgroup analysis of this trial, the biggest benefit in pCR was seen in patients with PD-L1-positive tumors. Presenting a simpler statistical analysis design compared to KEYNOTE-585, the results of the MATTERHORN trial in terms of its primary objective, EFS, are still awaited [17].

The German phase II/III AIO DANTE trial was designed to evaluate the efficacy and activity of atezolizumab (anti-PD-L1) in combination with perioperative FLOT. Before randomization, patients were stratified according to nodal stage (cN− vs. cN+), tumor site (GEJ type I vs. GEJ type II/III vs. gastric), and MSI status to receive four pre- and postoperative cycles of FLOT with atezolizumab followed by eight cycles of atezolizumab maintenance versus the conventional perioperative FLOT4 regimen [18].

The results of phase II, with 295 patients, showed a benefit in downstaging with the addition of atezolizumab to perioperative FLOT (ypT0 23% vs. 15%, *p* 0.044; ypT0-T2 61% vs. 48%, *p* 0.015; ypN0 68% vs. 54%, *p* 0.012). The complete histopathologic regression rates (pCR or TRG1a) were higher after FLOT plus atezolizumab (pCR/TRG1a 24% vs. 15%, *p* 0.032), especially in patients with a PD-L1 CPS ≥10 (pCR 33% vs. 12%) and MSI (pCR 63% vs. 27%) [18].

The phase III expansion of the trial will enroll 379 patients with locally advanced non-metastatic gastric and esophagogastric junction adenocarcinomas (≥cT2 and/or cN+) with at least one positive biomarker for immune response (MSI, PD-L1 CPS ≥ 1, TMB ≥ 10/MB or Epstein–Barr virus-positive, EBV+). Besides these inclusion criteria, a prospective biomarker study with serial circulating tumor DNA analysis will be performed. This phase III expansion will include, among other secondary endpoints, an OS and EFS analysis for the subgroups of patients with a PD-L1 CPS ≥ 5 and ≥10 and patients with MSI-high tumors as a secondary endpoint (NCT03421288) [19]. This preplanned secondary analysis may provide a better understanding of chemoimmunotherapy outcomes in this subgroup of patients.

Both of these trials (MATTERHORN and AIO-DANTE) showed similar improvements in pCR rates and downstaging with the addition of ICIs (approximately 10% increase) [17,18]. However, data on the primary endpoint of EFS are pending for both trials, and to date, no trial has shown an improvement in overall survival, so the current standard of care remains the same, without ICIs in the perioperative setting. Table 2 summarizes phase III clinical trials using immunotherapy combined with chemotherapy in perioperative gastric cancer treatment.

**Table 2:** Summary of phase III clinical trials involving immune checkpoint blockade in the perioperative treatment of gastric cancer.

Clinical Trial (Phase)	Setting	n	Treatment	Primary Endpoint	Outcomes	Reference
AIO DANTE (II/III)	Perioperative GEJ/GC ≥ T2 ou N+ (III: MSI, PD-L1 CPS ≥ 1, TMB ≥ 10/MB or EBV+)	674 (295 + 379)	FLOTx4 + ATEZOx4 → surg. → FLOTx4 + ATEZOx4 + ATEZOx8 Vs. FLOTx4 → surg. → FLOTx4	II:pCR/pTNM III:EFS	ypT0, 23% vs. 15% (*p* 0.044) ypT0-T2, 61% vs. 48% (*p* 0.015) ypN0, 68% vs. 54% (*p* 0.012) pCR/TRG1a 24% vs. 15% (*p* 0.032)	[18]
MATTERHORN (III)	Perioperative GEJ/GC >cT2N0-3 or N+	948	FLOTx4 + DURVAx2 → surg. → FLOTx4+ DURVAx2 + DURVAx10 vs. FLOTx4 → surg. → FLOTx4	EFS	pCR rate 19% vs. 7% (OR, 3.08, *p* < 0.00001) Combined pCR/near-pCR 27% vs. 14% (OR, 2.19; *p* < 0.00001)	[17]
KEYNOTE-585 (III)	Perioperative GEJ/GC ≥ cT3 or N+	1007	XP/FPx3 + PEMBROx3 → surg. → XP/FPx3 + PEMBROx3 + PEMBROx11 (Main cohort) or FLOTx4 + PEMBROx3 → surg. → FLOTx4+ PEMBROx3 + PEMBROx11 (FLOT cohort) vs. XP/FPx3/FLOTx4 → surg. → XP/FPx3/FLOTx4	pCR, EFS, OS (main cohort), safety (FLOT)	Main cohort: pCR 12.9% vs. 2.0% (*p* < 0.00001) Median EFS 44.4 vs. 25.3 mo (HR 0.81 *p* 0.0198, NS) Median OS 71.8 vs. 55.7 mo (HR, 0.86, 95% CI, 0.71 to 1.06)	[16]

ATEZO: atezolizumab; CCR: complete clinical response; EFS: event-free survival; FLOT: 5-fluorouracil, leucovorin, oxaliplatin, and docetaxel; FP: cisplatin and 5-fluorouracil; GEJ/GC: esophagogastric junction and gastric cancer; HR: hazard ratio; ICI: immune checkpoint inhibitor; MPR: major complete pathological response; n: number of patients randomized; NS: non-statistically significant; OR: odds ratio; pCR: pathological complete response; PEMBRO: pembrolizumab, DURVA: durvalumab; Surg.: surgery; TRG: tumor regression grade; XP: cisplatin and capecitabine.

Other questions are rising concerning the response to ICIs when combined with chemotherapy, as some preclinical studies have demonstrated a higher CD8+ T cell infiltration and an improved antitumor response when anti-PD-L1 is given before chemotherapy comparing to concomitant chemoimmunotherapy or subsequent anti-PD-L1 treatment [20]. In the phase II PANDA trial, patients received one cycle of atezolizumab monotherapy followed by four cycles of atezolizumab combined with chemotherapy. The results showed a major pathologic response in 14 of 20 patients (70%, 95% CI 46–88%), including 9 with pCR (45%, 95% CI 23–68%). At a median follow-up of 47 months, 13 of 14 responders to this perioperative strategy were alive and disease-free, and 5 out of 6 non-responders had a cancer-related death [21]. Even though these are promising results, this is a small trial and further randomized control studies are needed to clarify the best sequencing and combination strategy for ICIs in the perioperative setting. Other phase II clinical trials are exploring strategies with ICIs in the perioperative setting either before or concomitantly to chemotherapy with different ICI and chemotherapy backbones (GASPAR, MONEO, ICONIC, NEOSUMMIT 01, NCT0291816) [22,23,24,25,26]. Also, the use of induction ICIs followed by chemoimmunotherapy versus ICI monotherapy induction and doublet ICI induction therapy are being explored as perioperative strategies in clinical trials for locally advanced esophagogastric adenocarcinoma (IMAGINE) [27]. Table 3 summarizes phase II clinical trials involving ICIs in perioperative gastric cancer treatment.

#### MSI-High and EBV-Positive Tumors

Gastroesophageal adenocarcinomas present a very heterogeneous tumor immune microenvironment. These differences can possibly explain the variable responses to ICIs among TCGA classification groups [28]. EBV+ and MSI tumors with intense T-cell infiltrates and high PD-L1 expression show the best responses to ICI [29,30].

Although gastric cancer presents a low prevalence of MSI-H tumors, in the CLASSIC and MAGIC trials, MSI-H status was a favorable prognostic and potential negative predictive factor for neoadjuvant/adjuvant chemotherapy in resectable gastric cancer, with better survival with surgery only when compared to MSS patients (mOS not reached vs. 20.5 months; HR 0.42; *p* 0.09). MSI-H tumors even revealed a worse outcome with perioperative chemotherapy when compared to MSS patients (mOS 9.6 months vs. 19.5 months; HR 2.18; *p* 0.03) [11,31,32].

A large meta-analysis studying the role of MSI was performed on patients enrolled in the MAGIC, CLASSIC, ARTIST, and ITACA-S trials, and it confirmed MSI status as a robust prognostic factor and predictive biomarker for treatment response [33].

A post hoc analysis of studies on pembrolizumab in the first and second line for advanced gastric cancer revealed a superior benefit of pembrolizumab in the MSI-H gastric cancer population compared to other patients [34]. Despite the fact that perioperative chemotherapy is perceived as having a deleterious role in MSI-H patients, the role of ICIs in the perioperative setting is not clear, and to prove their potential benefit, several phase II trials were designed, as summarized in Table 4.

As mentioned above, EBV-positive gastric cancers also present a better response to ICIs, and they are also associated with better prognosis and fewer lymph node metastases, but they only represent 10% of gastric cancer patients, which makes it difficult to include this population in randomized clinical trials [29,30,35]. A few phase Ib/II trials are ongoing in the advanced stage for this specific population (EBVaGC-SYSUCC, 4-2017-0277, CIBI310Y101), and some of the more recent perioperative trials in gastric cancer also include these patients (AIO-DANTE, NCT03257163) [18,36,37,38,39,40].

Two phase II trials, NEONIPIGA and INFINITY, were designed to validate the use of a perioperative strategy with dual ICIs in patients with dMMR/MSI-H gastric cancer; both trials revealed high pCR rates after neoadjuvant treatment with anti–PDL1 and anti-cytotoxic T-lymphocyte–associated protein 4 (CTLA4) antibodies [41,42].

The NEONIPIGA phase II trial was a French study designed for the MSI-H population in which locally advanced esophagogastric junction/gastric cancer patients (cT2-4,Nx) received six cycles of neoadjuvant nivolumab (anti-PDL1) every 2 weeks and also neoadjuvant ipilimumab (anti-CTLA4) for two cycles 6 weeks apart, followed by nine cycles of adjuvant nivolumab post-surgery. Twenty-seven out of thirty-two patients completed all cycles of ICIs, and only six patients presented grade 3/4 adverse events (19%). Only twenty-nine patients underwent surgery because two patients with complete endoscopic response and tumor-free biopsies refused surgery. The primary endpoint was pCR, which was attainable in 17 patients (58.6%; 90% CI, 41.8 to 74.1). At final data reporting, there were no relapses or cancer-related deaths [41].

The phase II INFINITY trial investigated the activity and safety of the combination of tremelimumab (anti-CTLA4) and durvalumab (anti-PDL1) as a neoadjuvant (Cohort 1) or definitive (Cohort 2) treatment for dMMR/MSI-H and EBV-negative locally advanced esophagogastric junction/gastric cancer patients (≥cT2 or N+) [42]. Patients in both cohorts received a single high dose of tremelimumab and concomitant durvalumab monthly for three cycles, completing neoadjuvant treatment in 12 weeks. Then, all patients performed new exams with ctDNA, CT, PET-CT, and endoscopic ultrasound with biopsy. In Cohort 1, patients proceeded to surgery followed by standard follow-up, and in Cohort 2, those patients with complete response in evaluation exams would follow nonoperative management with a close surveillance program (every 12 weeks), and patients without complete response proceeded to surgery and standard follow-up as patients in Cohort 1.

The primary endpoint was pCR, which occurred in 60% of patients, all of them with negative ctDNA pre-surgery. Also, major complete pathological response was observed in 80% of patients. Patients with larger tumors at diagnosis, like T4, presented a pCR of 17% (1 in 6 patients) and T2-3 tumors a pCR of 89% (8 in 9 patients, *p* 0.011), whereas no correlation was found with N status at diagnosis. PDL-1 CPS was not associated with outcomes and TMB had a non-significant trend of correlation with pCR. All patients underwent surgery, except for two patients with complete response that refused surgery. There were no severe adverse events reported. Cohort 2 enrollment is ongoing [42].

The results of these two trials are very promising and their high pCR rates have led to the consideration of ICIs as a preferred approach for the perioperative management of dMMR/MSI-H GEJ/GC patients. Also, the role of surgery in this population is being questioned, and a potential omission of surgery needs to be studied in randomized clinical trials.

For now, international guidelines do not give any consideration to MSI status as a determinant factor for a different perioperative approach than the standard FLOT4 perioperative regimen recommended for EGJ/GC patients [43].

**Table 4:** Summary of clinical trials involving immune checkpoint blockade in the perioperative treatment of MSI gastric cancer.

Clinical Trial (Phase)	Setting	n	Treatment	Primary Endpoint	Outcomes	Reference
NEONIPIGA (II)	Perioperative GEJ/GC MSI cT2-4,Nx	32	NIVOx6 + IPIx2 → surg. → NIVOx9	pCR	pCR 58.6% (17/29 [90% CI, 41.8 to 74.1])	[41]
INFINITY (II)	Perioperative GEJ/GC MSI ≥cT2 or N+	18/13	Cohort 1: TREMEx1 + DURVAx3 → surg. Cohort 2: TREMEx1 + DURVAx3 → surg. vs. nonoperative management if CRR	Cohort 1: pCR, ctDNA(-) Cohort 2: CRR 2y	Cohort 1: pCR 60% (9/15) and MPR 80%. All pts with pCR had ctDNA (−) Cohort 2: ongoing	[42]
IMHOTEP-5 (II)	Perioperative EG, CRC, EC, OC MSI	120	PEMBROx1-2 → surg. → PEMBRO 1y	pCR	pCR 38.9% (40.7%, 25.0%, 0.0% and 85.7% in CRC, EG, EC and OC, respectively)	[44]
EA2212 (II)	Perioperative GEJ/GC MSI	240	ATEZO + ChT (FLOT/mFOLFOX/XELOX)x3-4 → surg. → ATEZO + ChTx3-4 → ATEZOx9 vs. ATEZOx3 → surg. → ATEZOx9	EFS	Recruiting	[40]
NCT03257163(II)	Perioperative GC MSI/EBV(+)/PD-L1 CPS ≥1, T2-4, N0-3	33	PEMBROx2 → surg. → CAPE + PEMBROx1 → CRT (45 Gy/25 fr) with CAPE + PEMBROx5 → PEMBRO 1y	DFS	Recruiting	[45]

ATEZO: atezolizumab; CAPE: capecitabine; XELOX: capecitabine and oxaliplatin; CRC: colorectal cancer; CRR: complete response rate; CRT: chemoradiation therapy; ctDNA: circulating tumor DNA; DURVA: durvalumab; EC: endometrial cancer; EFS: event-free survival; EG: esophagogastric cancers; FLOT: 5-fluorouracil, leucovorin, oxaliplatin, and docetaxel; GEJ/GC: esophagogastric junction and gastric cancers; pCR pathological complete response; mFOLFOX: modified 5 FU and oxaliplatin; MPR: major complete pathological response; MSI: microsatellite instability; n: number of patients randomized; NIVO: nivolumab; OC: other cancer; PEMBRO: pembrolizumab; surg.: surgery; TREME: tremelimumab.

The phase II IMHOTEP trial studied the perioperative immunotherapy strategy with pembrolizumab in several dMMR/MSI-H tumors; among them were patients with esophagogastric cancers (n = 21) that obtained a pCR of 25% with only 1–2 cycles of neoadjuvant pembrolizumab. Five patients chose not to proceed to surgery due to a complete clinical response. Despite recording a lower pCR than that reported in other studies, the results are optimistic, with a good toxicity profile. So, we can hypothesize that with long preoperative treatment duration with ICIs, a greater pCR may be obtained. Further results are awaited, and more investigatory work is required to assess the optimal timing and duration of the perioperative ICI strategy for MSI-H tumors [44].

There are several ongoing studies assessing multimodal strategies using a combination of chemotherapy and ICIs in MSI-H tumors versus ICIs only (EA2212), as well as trimodal perioperative strategies with a combination of chemotherapy, ICIs, and post-surgery radiotherapy in patients with MSI-H, EBV(+), or PDL1 CPS ≥ 1 gastric cancers (NCT03257163) [40,45].

### 3.2. Targeted Therapies

During the past decade, a deeper understanding of the molecular behavior and subtypes of gastric cancer led to the development and integration of new targeted agents in the palliative setting of gastric cancer, showing great benefits so far. In the perioperative setting, targeted therapy has just begun to take its first steps, with no approved agents to this day, but with some promising results for the near future.

#### 3.2.1. HER-2

Human epidermal growth factor-2 (HER-2) is a transmembrane tyrosine kinase receptor and an epidermal growth factor receptor (EGFR) whose activation results in uncontrolled cell growth and proliferation [46]. HER-2 overexpression in gastric cancer was first described in 1986 [47] and it is estimated to be present in approximately 10–30% of gastric cancers [7]. HER-2 overexpression is associated with worse overall prognosis and poor response to chemotherapy [48]. Trastuzumab, a monoclonal antibody (mAb) consisting of a humanized immunoglobulin G1, inhibits the HER-2 pathway by binding the receptor’s subdomain IV and by inducing antibody-dependent cell-mediated cytotoxicity [49,50]. After its initial role in HER-2-positive breast cancer, trastuzumab proved to also increase overall survival in metastatic gastric cancer when associated with first-line chemotherapy [51], which is now the standard initial treatment for HER-2-positive advanced gastric cancer, in combination with pembrolizumab [14]. Several other HER-2 agents are currently under study in ongoing trials in the palliative setting [52,53].

It is also important to note that HER-2 upregulates PD-L1 expression as an immune resistance mechanism, which is the basis for the ICI and anti-HER-2 combination in the KEYNOTE-811 trial. This trial showed statistically significant and clinically meaningful improvement in OS, PFS, and ORR in all patients with unresectable or metastatic HER2+ gastric/GEJ adenocarcinoma, especially in patients with dual HER2 positivity and PD-L1 CPS ≥1 [14].

In resectable gastric cancer, HER-2 also proved to be an independent adverse prognostic factor, associated with lower survival rates regardless of tumor stage and of HER-2 heterogeneity [54]. Following this lead, anti-HER-2 agents started to integrate clinical trials, also in the perioperative setting, as summarized in Table 5.

HER-FLOT was a multicenter phase II single-arm study that tested the addition of trastuzumab to perioperative chemotherapy with the FLOT protocol in HER-2-positive gastroesophageal cancer staged ≥cT2, any N, M0. Trastuzumab was continued for nine more cycles after chemotherapy suspension. The study met its primary endpoint of reaching a pCR rate above 20% (21.4%), with 25.0% nearly pCR, against 12.8% pCR and 16.7% nearly pCR in the original FLOT4-AIO trial. Median DFS was 42.5 months and 3-year OS was 82.1% [10,55].

NEOHX was a Spanish multicenter phase II study that also evaluated the addition of trastuzumab to perioperative chemotherapy using XELOX protocol. The primary endpoint was 18-month DFS and was 71% (95% CI 53–83%), and median OS was 79.9 months, 2.5 years more than in the FLOT4-AIO trial [10]. The pCR rate was 9.6% and the pathological downstaging rate was 58% [56].

A French retrospective observational study, AGEO, also evaluated the addition of trastuzumab to perioperative chemotherapy with either FOLFOX (5-fluorouracil/oxaliplatin) or TFOX (docetaxel/oxaliplatin). The results were encouraging, with rates of major and complete pathological response of 42% and 10%, respectively, and an 18-month DFS of 80.4% (95% CI 68.9–93.8%) and a 2-year OS of 89.0% (95% CI 79.5–100.0%) [57].

A more recent phase II single-arm Chinese trial tested the association of trastuzumab and tislelizumab with the Asian DOS (docetaxel, oxaliplatin and S-1) protocol for 3 neoadjuvant and 12 adjuvant cycles. The study attained a pCR of 47.1% and a major pathological response of 70.6%, with a 1-year overall survival rate of 91.7; the median has not yet been reached [58].

Pertuzumab is a humanized mAb that binds HER-2 subdomain II, preventing its dimerization with other HER family receptors, precluding its downstream signaling and consequent tumor proliferation [59]. Regarding the promising results of associating trastuzumab with perioperative chemotherapy, two subsequent studies were developed to evaluate the addition of both trastuzumab and pertuzumab to this strategy.

PETRARCA was a multicenter double-arm phase II/III study that evaluated the addition of trastuzumab and pertuzumab versus placebo to perioperative FLOT. The pCR rate was 35%, significantly higher than the 12% in the comparative arm (*p* = 0.019), meeting the study’s primary endpoint of reaching a pCR > 25%. DFS and OS at 24 months were, respectively, 70% vs. 54% (HR 0.58, *p* = 0.14) and 84% vs. 77% (HR 0.56, *p* = 0.24), and the medians have not been reached yet [60]. Despite these compelling results, the study was prematurely terminated after the obsolete benefits of double HER-2 blockade shown in the metastatic setting in the JACOB trial [61].

The phase II intercontinental EORTC trial INNOVATION evaluated the association of trastuzumab with or without pertuzumab with perioperative chemotherapy, in a ratio of 2:2:1, in patients with stage Ib-III HER-2-positive gastric or GEJ cancer, with the initial recruited patients being treated with CF/CX (cisplatin with 5-fluorouracil or capecitabine), and the latter with FOLFOX, XELOX, or FLOT [62]. Before the early termination of recruitment due to slow accrual, 161 patients were included. Major pathological response was observed in 23.3% of patients treated with only chemotherapy, in 37.0% when trastuzumab was added (*p* = 0.378), and only 26.4% with trastuzumab and pertuzumab (*p* = 0.099 when compared to the chemotherapy arm), not fulfilling the study’s primary endpoint. Moreover, 70% of patients suspended treatment due to high toxicity with double HER-2 blockade, which is one of the most likely explanations for the disappointing response rates to the combination when compared to trastuzumab monotherapy [63].

KN026 (or anbenitamab) is a bispecific antibody combining components of both trastuzumab and pertuzumab in its structure, thus targeting two different epitopes of HER-2. After promising results in the metastatic setting [64], it is currently being evaluated in the perioperative setting in a Chinese phase II study, in combination with KN046, also a bispecific antibody targeting PD-L1 and CTLA-4, both alone and in combination with XELOX (NCT06023758). Its primary endpoint is the pCR rate and preliminary data are awaited.

Trastuzumab deruxtecan is an antibody–drug conjugate composed of the anti-HER-2 monoclonal antibody trastuzumab and a cytotoxic payload, SN-38 or deruxtecan, which inhibits topoisomerase I. It has demonstrated major survival benefits, initially in HER-2-positive breast cancer [65]. After positive results in HER-2-positive metastatic gastric cancer also [52,66], it is now being evaluated in the neoadjuvant setting in a Japanese phase II study, EPOC2003 [67] (NCT05034887), in HER-2-positive and HER-2-low gastric or gastroesophageal junction cancer, given as three cycles every 3 weeks prior to gastric surgery. According to the latest reported data in HER-2-positive patients, the study has not yet met its primary outcome of reaching a major pathological response rate of 20%, as it was only documented in 14.8% of patients so far, with 3.7% of pCR. This reveals that T-DXd is probably ineffective as a single agent for the neoadjuvant treatment of HER-2 gastric cancer. This agent is also being evaluated in the adjuvant setting, in cases of residual disease in liquid biopsy after preoperative FLOT, in the Italian phase II study TRINITY [68] (NCT06253650), whose results are awaited.

Disitamab vedotin, also known as RC48, is also an antibody–drug conjugate that combines hertuzumab, a new monoclonal antibody targeting subdomain IV of HER-2 in a different epitope than trastuzumab, with monomethylauristatin E (MMAE), also known as vedotin, a cytotoxic agent that acts by inhibiting tubuline polymerization during cell division [69]. After promising results in phase I trials in the metastatic setting [70], disitamab vedotin is currently being tested in the early setting in combination with chemotherapy and immune checkpoint inhibitors. A phase II Chinese trial is testing its association with neoadjuvant S-1 and camrelizumab, an anti-PD-1 ICI, in HER-2-positive cases, with the latest reports showing promising results, with a major pathological response rate of 50% and a pCR of 31%. Median DFS and OS have not yet been reached [71]. A phase II study is currently recruiting patients in China with HER-2-positive or -low gastric and gastroesophageal junction cancer to undergo perioperative XELOX combined with toripalimab, an anti-PD-1 monoclonal antibody, with or without disitamab vedotin, followed by 1 year of maintenance with toripalimab, with pCR rates as the primary endpoint [72] (NCT06155383). A very similar phase II trial is planned to open soon, also in China, testing sintilimab, another anti-PD-1 ICI, also in combination with XELOX and disitamab vedotin (NCT06227325).

Other HER-2-targeted agents, such as margetuximab [73], tucatinib [74], neratinib (NCT06109467), zanidatamab [53], and ARX788 [75], are being tested in the palliative setting of HER-2-positive gastric cancer and have not yet been included in trials in the perioperative setting. Some agents, such as trastuzumab emtansine [76], showed disappointing results in the palliative setting and were therefore not pursued in the curative setting.

**Table 5:** Summary of clinical trials integrating HER-2-targeted agents in the perioperative treatment of gastric cancer, with published results.

Clinical Trial (Phase)	HER-2-Targeted Agent	n	Treatment	Outcomes	Status	Reference
HER-FLOT (II)	Trastuzumab	56	FLOT + Tx4 → surg. → FLOT + Tx4 → Tx9	pCR 21.4% R0 resection rate 92.9% mDFS 42.5 m; OS-3y 82.1%	Published	[55]
NEOHX (II)	Trastuzumab	36	XELOX + Tx3 → surg. → XELOX + Tx3 → Tx12	pCR 9.6% R0 resection rate 90% DFS 18 mo 71.0%; mOS 79.9 mo	Published	[56]
AGEO (retrosp)	Trastuzumab	48	FLOT/FOLFOX + Tx4 → surg. → FLOT/FOLFOX + Tx4	MPR 42%, pCR 10% DFS 18 mo 80.4% (95% CI 68.9–93.8) OS 2y 89.0%	Published	[57]
NCT04819971 (II)	Trastuzumab	12	Tislelizumab + Tx1 → DOS + tislelizumab + Tx3 → surg. → DOS + tislelizumab + Tx3 → Tislelizumab + Tx6	MPR 57.1%, pCR 42.9% R0 resection rate 100% mDFS and mOS not reached	Published	[58]
PETRARCA (II)	Trastuzumab ± pertuzumab	81	FLOT ± T+Px4 → surg. → FLOT ± T+Px4 → T + Px9	pCR 35% vs. 12% (*p* 0.019) ypN0 68% vs. 39% R0 resection rate 93% vs. 90% DFS 2y 70% vs. 54% (HR 0.58, *p* = 0.14) OS 2y 84% vs. 77% (HR 0.56, *p* = 0.24)	Terminated	[60]
INNOVATION (II)	Trastuzumab + pertuzumab	161	FP/XELOXx3/FOLFOX/FLOTx4 ±T ± Px3 → surgery → /FP/XELOXx3/FOLFOX/FLOTx4 ± T ± Px3 → T ± Px17	MPR 23.3% (ChT) vs. 37.0% (+T) vs. 26.4% (+T+*p*) (*p* = 0.378/0.099) R0 resection rate 83.9% (ChT) vs. 90.3% (+T) vs. 85.9% (+T+P)	Terminated	[63]
EPOC2003 (II)	T-DXd	27	T-DXd x3 → surg.	pCR 3.7% No data on DFS/OS	Recruiting	[67]
ChiCTR2300075446 (II)	Disitamab vedotin	28	S1 + camrelizumab ± DV x3 → surg.	MPR 50%; pCR 31% DFS/OS not reached	Recruiting	[71]

A: adjuvant; ChT: chemotherapy; DFS: disease-free survival; DOS: docetaxel, oxaliplatin, and S-1; DV: disitamab vedotin; FLOT: 5-fluorouracil, leucovorin, oxaliplatin, and docetaxel; FP: 5-fluorouracil and cisplatin; GC: gastric cancer; GEC: gastroesophageal cancer; GEJC: gastroesophageal junction cancer; HR: hazard ratio; LAGC: locally advanced gastric cancer; mo: months; MPR: major pathological response rate; OS: overall survival; P: pertuzumab; pCR: pathological complete response rate; PO: perioperative; surg.: surgery; T: trastuzumab; T-DXd: trastuzumab deruxtecan.

#### 3.2.2. VEGF

Angiogenesis is well recognized to be essential for cancer growth, invasion, and metastasis, and it is considered a hallmark of cancer [77]. Vascular endothelial growth factor (VEGF), a family of homodimeric glycoproteins, was first described in 1989 [78] and was found to regulate vascular permeability, angiogenesis, and lymphogenesis through binding one of the VEGF receptors (VEGFR1, 2, and 3) and activating its downstream pathway [79]. VEGF pathway hyperactivation has been described in several different cancer types, including gastric cancer [80]. Several anti-angiogenic agents have been tested in advanced gastric cancer treatment, but so far, only ramucirumab has proved its value in association with single-agent chemotherapy, and it is the only anti-VEGF agent approved in this setting [81,82].

Bevacizumab, a monoclonal antibody that targets VEGF-A, was first evaluated in the perioperative treatment of gastric cancer in a British phase II/III trial, ST03, in combination with ECX (epirubicin, cisplatin, and capecitabine). The study revealed no benefit in the addition of bevacizumab, with similar 3-year OS (48.1% against 50.3%) and more surgical complications [83].

RAMSES/FLOT7, a phase II/III trial, tested the addition of ramucirumab, a monoclonal antibody targeting VEGF receptor-2, to perioperative FLOT in HER-2-negative gastric/GEJ cancer [84]. The most recent results revealed a higher R0 resection rate, with 96% against 82% without ramucirumab, as well as a slightly improved median DFS (32 against 21 months) but similar major pathological response rates (26% against 29%) and similar median OS (46 versus 45 months), with higher rates of postoperative complications [85].

DRAGON-IV, a Chinese phase II/III trial, is testing the addition of apatinib, also known as rivoceranib, a selective VEGF receptor-2 inhibitor, to perioperative SOX and camrelizumab followed by apatinib and camrelizumab maintenance. Preliminary results indicated encouraging pCR and MPR rates of 18.3% and 51.1%, respectively, with apatinib and camrelizumab association, significantly superior to the rates of 5.0% and 37.8% achieved with only SOX, with no increase in postoperative complications (27.7% against 30.1%) [86] (NCT04208347). SPACE-neo is another ongoing phase II trial in China which is also testing the association of apatinib with SOX and camrelizumab with a merely preoperative regimen; the results have not yet been disclosed [87] (ChiCTR2100049305).

EPOC2001, a phase II trial currently recruiting in China, is testing the association of the pan-VEGF receptor inhibitor lenvatinib with perioperative FLOT added to pembrolizumab, which is maintained for 11 more cycles (NCT04745988). Preliminary results showed major pathological response and pCR rates of 47% and 22%, respectively, still below the predefined threshold of 30% pCR. So far, no dose-limiting toxicity has been reported [88]. Further results are expected.

Surufatinib is a novel small-molecule inhibitor that targets VEGF receptors 1, 2 and 3, FGFR, and also immune evasion through the inhibition of macrophage colony-stimulating factor-1 (CSF-1) [89]. This new drug is being evaluated in a Chinese phase II trial in the treatment of resectable gastric cancer in association with perioperative SOX and sintilimab (NCT06447636), and results are awaited.

Finally, a phase II trial is evaluating neoadjuvant treatment with SOX associated with fruquintinib, a potent and highly selective tyrosine kinase inhibitor that targets VEGF receptors 1, 2, and 3 [90] (NCT05122091), with no results available yet. Table 6 summarizes the results of clinical trials involving anti-VEGF agents in perioperative gastric cancer treatment.

#### 3.2.3. Emerging Biomarkers

Regarding recent progress in the molecular characterization of gastric cancer, new targets are being tested in the treatment of gastric cancer. Claudin 18.2 is a tight junction protein expressed exclusively in normal gastric cancer cells and it was discovered to be retained and expressed in 58 to 74% of all gastric cancers [91], with 38% expression reported in resectable cases [92]. It became an attractive therapeutic target, and clinical trials with the anti-claudin 18.2 mAb zolbetuximab in patients who express this protein are revealing impressive results in the metastatic setting [93], so its inclusion in perioperative treatment trials is expected.

Fibroblast growth factor receptor (FGFR) is a tyrosine kinase present in the cell membrane whose somatic mutations are frequently linked to carcinogenesis in several tumor types. FGFR-2 was found to be amplified in 10% of gastric cancers and seems to correlate with a poorer prognosis [94]. Its inhibition through the mAb bemarituzumab showed favorable results in a phase II trial [95] and is currently being tested in the advanced setting in a phase III trial (NCT05052801), but its inclusion in the early setting is not expected soon.

### 3.3. Role of Radiotherapy in Resectable Gastric Cancer

As reviewed earlier, perioperative chemotherapy is the standard of care for localized gastric cancer (stage IB-III) according to international guidelines [43]. Multimodal strategies were studied in the perioperative setting and the role for radiotherapy was explored in three phase III trials—the CRITICS trial from the Netherlands and additionally in the Korean studies ARTIST and ARTIST II.

In the CRITICS trial, patients were randomized to receive chemotherapy with three cycles of ECX/EOX preoperatively and postoperatively or preoperative chemotherapy and postoperative chemoradiation (45 Gy/25 fractions) with concomitant capecitabine and cisplatin (XP). The results showed no benefit in survival for postoperative chemoradiotherapy when compared to perioperative chemotherapy (mOS 61.4 months vs. 43 months, HR 1.01, *p* 0.90) in patients with resectable gastric cancer treated with adequate preoperative chemotherapy and surgery [96].

ARTIST and ARTIST II studied adjuvant alternatives in the Asian population. In this population, upfront surgery followed by adjuvant treatment with chemotherapy and radiotherapy is the most frequently adopted strategy, in opposition to Western countries, where perioperative FLOT4 is standard practice. In these trials, patients who underwent surgery and D2 lymph node dissection were randomly assigned to adjuvant chemotherapy (XP) for six cycles or chemoradiation (CRT) with two cycles of XP plus radiotherapy with capecitabine followed by two more cycles of XP. The addition of CRT to XP chemotherapy did not significantly reduce recurrence in this trial. In the subsequent trial (ARTIST-II), which included only patients with node-positive status, postoperative CRT did not add any benefit in recurrence; as a result, it is not recommended as standard practice. However, for patients with gastric cancer who have not received preoperative chemotherapy and with inappropriate D2 lymphadenectomy, adjuvant CRT can be considered according to international guidelines [43,97,98].

The role of preoperative radiotherapy was also considered for many reasons: its better tolerability when compared to chemotherapy, which ensures a higher percentage of patients completing preoperative treatment; possible tumor downstaging and improved R0 resection rates; and lastly, its important role as the standard of care for esophageal cancer. Recently, the phase III ESOPEC trial for esophagus and EGJ adenocarcinoma showed the superiority of the FLOT regimen when compared with neoadjuvant CRT according to the CROSS protocol, with a median overall survival improvement of 29 months, reinforcing perioperative FLOT plus surgery as the standard treatment in esophageal and EGJ adenocarcinomas [99].

The phase III TOPGEAR trial was the first trial designed to clarify the role of adding preoperative CRT to perioperative chemotherapy in patients from centers all over the world undergoing surgery for resectable gastric cancer. In this trial, patients with resectable gastric or EGJ adenocarcinoma (Siewert type II ≤ 2 cm esophageal involvement and Siewert type III) cT3-4 or N+ were randomized to perioperative chemotherapy with FLOT or ECF alone or plus preoperative chemoradiotherapy (45 Gy with 5-fluorouracil).

The results, presented in September this year at the European Society for Medical Oncology Congress, showed that adding preoperative CRT to perioperative chemotherapy did not affect the rates of curative resection, and it was not associated with increased toxicity or surgical morbidity; although it resulted in a higher pCR rate (16.8% vs. 8.0%, *p* < 0.0001) and tumor downstaging (ypT0, ypTis 16.5% vs. 7.3%, *p* < 0.001; ypN negative 54.1% vs. 42.3%, ypN positive 45.9% vs. 57.7%, *p* < 0.01), it did not improve overall survival (mOS 46.4 months vs. 49.4 months; HR 1.05, 95 CI 0.83 to 1.31, *p* = 0.70), with a 3-year OS of 55.1% vs. 57.7% and a 5-year OS of 44.4% vs. 45.7% for CRT compared to perioperative chemotherapy [100].

These trials provide evidence that there is no place for the addition of CRT to the perioperative treatment of gastric cancer, and this practice may need to be reconsidered, on a case-to-case basis, in centers where it remains standard practice.

## 4. Discussion

In recent years, with deeper knowledge about tumor biology and biomarkers for gastric cancer, treatment approaches in the perioperative setting are evolving from conventional chemotherapy to biomarker-driven treatment strategies. However, studying new strategies for perioperative treatment in gastric cancer is not easy because of its tumoral heterogeneity and diverse genomic subtypes. Also, as a result of biomarkers’ rarity, most clinical trials include small sample sizes, and consequently, small differences and subgroup effects may be underestimated, making it difficult to develop effective targeted therapies [43].

Nevertheless, further evidence is needed to add these agents to the perioperative setting. The published results from the three randomized phase III trials mentioned above are still immature. KEYNOTE-585 is the trial presenting the most mature data on EFS and pCR, with superior outcomes for chemoimmunotherapy. Still, unfortunately, these positive results were not statistically significant, and the same happened with the recent report for overall survival [16]. One of the reasons pointed out by most experts for these disappointing results is the complex statistical analysis of the trial, while others point out the inferior chemotherapy regimen given to 80% of patients enrolled [16]. Further results from the MATTERHON and AIO DANTE trials may address some of these limitations.

Ahead of the previously mentioned trials is AIO DANTE, with a phase III expansion for patients with locally advanced gastric and esophagogastric junction adenocarcinoma with at least one positive biomarker for immune response, a subpopulation that showed a distinct and promising benefit from ICIs on pCR. The preplanned OS and EFS analysis for the subgroups of patients with PD-L1 CPSs ≥5 and ≥10 and patients with MSI-high tumors will shed light on future biomarkers for patient selection [18]. The future seems bright for ICIs in the perioperative setting, with a statistically strong improvement of 10% in pCR in both MATTERHORN and AIO-DANTE [17,18]. However, data on primary endpoint EFS are still pending for both trials and, to date, no trial has shown an improvement in overall survival.

We should also be aware that in KEYNOTE-585, a better pCR with chemoimmunotherapy did not translate into a positive result in overall survival. This should make us question whether pCR is a robust surrogate endpoint for survival in non-chemotherapy trials like it was in the FLOT4 trial, in which a higher pCR translated into a survival benefit [10]. Also, none of the chemoimmunotherapy trials showed the same pCR reported in the FLOT4 trial, not even the chemoimmunotherapy arm. In other tumors, such as in early-stage triple-negative breast cancer and non-small-cell lung cancer, a strong association between pCR and EFS was shown, and in the exploratory analysis, the EFS benefit was independent of pCR in the ICI arm, showing a potential additional role for the adjuvant part of the ICI perioperative strategy [101,102,103,104,105]. However, further data from MATTERHORN and DANTE will be important to discuss this potential benefit. Another interesting surrogate for survival would be the postoperative pathological lymph node stage, as suggested in the post hoc analysis results from the MAGIC trial; it should be addressed as a potential surrogate marker for survival in further trials [106]. In conclusion, pCR as a prognostic marker is not as established in other settings as it is for chemotherapy trials, and the survival results from MATTERHORN and DANTE may help us conclude whether pCR is a reliable predictor of survival for perioperative chemoimmunotherapy also.

Within this revision of perioperative strategies, two populations stand out for future tailored treatment approaches: the MSI-high population and the HER-2-positive population. Both HER-2 positivity and MSI-H are established prognostic and predictive biomarkers for treatment response in GC [31,32,54,107,108].

The MSI-high population showed impressive results under perioperative treatment with ICIs in some phase II trials, such as NEONIPIGA and INFINITY, mentioned above. Even though these trials showed promising results, with pCR rates of approximately 60%, they are small trials and further randomized control studies are needed to clarify the best sequencing and combination strategy for ICIs in the perioperative setting [41,42]. Also, the role of surgery in this population is being questioned and surely needs more investigatory work; the Cohort 2 results of INFINITY may give us a glimpse of this subject [42].

The phase III expansion of AIO-DANTE included not only MSI-H tumors but also other tumors with positive biomarkers for immune response, as already mentioned [18]. We know that the results for these subpopulations in other trials were very promising, with better results for pCR in the subgroup analysis for PDL1-positive tumors in the MATTERHORN trial and the subgroup analysis for EFS and OS in KEYNOTE-585, confirming a higher benefit in MSI-H patients and in patients with tumors with CPS > 10 [16,17]. Further results on EFS and OS from MATTERHORN and results from the phase III expansion of AIO-DANTE are awaited. We hope these studies may impact the design of future trials in this specific population and possibly change standard practice for MSI-high tumors.

One lesson we learned from perioperative trials with chemoimmunotherapy is that biomarkers for response to ICIs need to be further explored for patient selection in future clinical trials, as we know that patient selection is key to obtain better outcomes from multimodal strategies, including ICIs, in the perioperative setting. New biomarkers for response to ICIs, namely, PDL-1 expression and TMB, have been reported in the advanced setting, both of them associated with better prognosis [109,110]. PDL-1 CPS was proved to be the second strongest predictive factor for ICI benefit [111]. One of its limitations is establishing a positive threshold for PD-L1 that guarantees the greatest benefit from ICI treatment. In advanced GC, a PD-L1 CPS ≥10 demonstrated better responses to pembrolizumab, and for nivolumab, a CPS ≥5 was sufficient for OS and PFS benefits [13,15]. As for TMB, in later lines of treatment for advanced/metastatic solid tumors, the KEYNOTE-158 trial showed promising results with pembrolizumab in tumors with a TMB-H ≥ 10 mut/Mb and included 24 GC patients (10.3%) [9]. In an exploratory analysis of the KEYNOTE-061 trial, TMB was found in 13–17% of patients with G/GEJ cancer, and an association with response to ICIs was reported, with better ORR and mOS in patients with a TMB ≥10 mut/Mb even after excluding MSI-H patients from the analysis [110]. In further study designs in the perioperative setting, it is necessary to use ICIs according to PD-L1 CPSs and the TMB cutoff.

As for HER-2-positive tumors, accounting for 10–30% of gastric cancer cases [7], one of the main concerns is the heterogeneity of this biomarker within the tumor, which may restrict patient selection and accrual for clinical trials. HER-2 is an adverse prognostic factor, both in the advanced and in the early disease setting, so targeting this molecular alteration is crucial [48,54]. Similarly to what was seen in the metastatic setting [14,51], current evidence shows that the association of trastuzumab with standard perioperative chemotherapy might provide compelling response rates, with pCR rates of approximately 10 to 20% and survival benefits that surpass the results of the currently standard FLOT-AIO4 trial, as demonstrated in the HER-FLOT and NEOHX trials [10,55,56,57]. Pertuzumab in addition to perioperative treatment was not demonstrated to add any benefits on top of trastuzumab, revealing even lower response rates and significantly higher toxicity when compared to the addition of only trastuzumab to chemotherapy [60,63]. On the other hand, as was the case in the metastatic setting, adding ICIs to trastuzumab in the perioperative setting was revealed to be beneficial, with increased pCR and major pathological response rates and high survival rates in a single Asian phase II study [58], the final results of which are yet to be confirmed. Other classes of HER-2-targeted drugs, such as bispecific antibodies, are currently being studied in phase II trials with favorable safety profiles and promising anti-tumor activity [64].

Antibody–drug conjugates are also emerging as a new class of drugs in the perioperative gastric cancer landscape, for now only in HER-2-positive disease. Trastuzumab deruxtecan has only been tested as a monotherapy in the neoadjuvant setting, with disappointing results, showing that it is probably not effective as a monotherapy [67]. As for disitamab vedotin, it did reveal promising antitumoral activity in combination with perioperative chemotherapy (S-1) and ICIs (camrelizumab), with pCR rates reaching more than 30% [71] and with survival benefits yet to be disclosed, substantiating its ongoing evaluation in combination with further chemotherapy and immunotherapy agents in the perioperative setting [72].

In summary, evidence on early HER-2-positive gastric cancer seems to point in the direction of a combination of chemotherapy, single HER-2 inhibition, either with trastuzumab or disitamab vedotin according to the current trials, with the eventual addition of ICIs. Notably, data from phase III trials are still lacking in this setting.

With regards to VEGF pathway inhibition, in the phase II/III trial DRAGON-IV, only apatinib showed an advantage when combined with perioperative chemotherapy (SOX) and ICIs (camrelizumab), independently of VEGF expression, demonstrating significant response rates, with a pCR of 18% [86]. No significant response or survival benefits were reported with bevacizumab or ramucirumab in the ST03 and RAMSES/FLOT7 trials and postsurgical complications were significantly higher when these agents were added to perioperative chemotherapy [83,85], unlike what happened with apatinib in DRAGON-IV [86]. Regarding the diverging data on the role of VEGF inhibition in perioperative gastric cancer treatment, new trials using innovative anti-VEGF agents are ongoing, and more elucidating data are awaited.

With reference to future targets and new treatment strategies, we would like to highlight Claudin 18.2 and FGFR-2, which are currently being tested in the metastatic setting [93,95]. However, we cannot forget that biomarker expression may evolve over time and within the same tumor which may translate into tumoral spatial and temporal heterogeneity; also, promising results in the advanced setting may not be reproducible in the perioperative setting [112].

**Figure 1:**
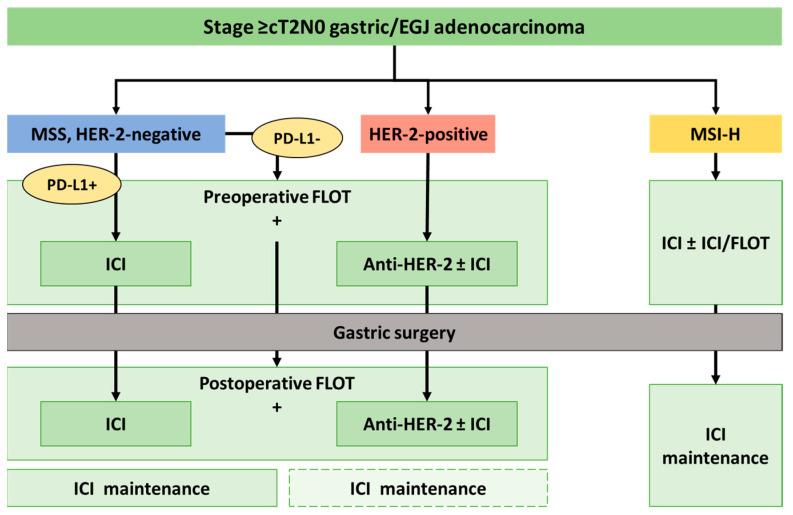
Schematic algorithm proposed by the authors for perioperative treatment of gastric cancer relying on biomarker-driven strategies and current evidence (image source: elaborated by the authors). EGJ: esophagogastric junction; HER-2: Humanized Epidermal Growth Factor Receptor-2; ICI: immune checkpoint inhibitor; MSS: microsatellite stable; MSI-H: microsatellite instability-high; PD-L1: Programmed Cell Death Ligand-1.

Figure 1 illustrates a proposed biomarker-driven algorithm, elaborated by the authors, for the management of locally advanced gastric cancer. The algorithm incorporates perioperative strategies based on specific biomarkers of interest, as previously discussed, including HER-2, microsatellite status (MSS or MSI-H), and PD-L1. The treatments vary depending on the patients’ biomarker profile. We would recommend that patients be initially assessed for microsatellite status and HER-2 expression. If a patient is HER-2-positive, anti-HER-2 therapy should be added to the standard chemotherapy regimen, eventually with the addition of ICIs. In MSI-H, there is a benefit in including immunotherapy in perioperative treatment, but more evidence is needed to determine whether it should be given in monotherapy or in combination with standard chemotherapy or with a second ICI. For MSS HER-2-negative tumors, the authors propose an evaluation of PD-L1 expression. For PD-L1-positive patients, ICI should be included in the perioperative strategy alongside FLOT. For triple-negative patients (HER-2-negative, PD-L1-negative, and MSS), the treatment should follow the current standard of care with the FLOT regimen. After surgery, patients should complete their perioperative treatment plan and proceed with maintenance therapy, which consists solely of biomarker-driven therapies (ICIs and/or anti-HER-2). Our increasing understanding of molecular subtypes and biomarkers will likely continue revolutionizing the treatment of gastric cancer.

## 5. Conclusions

Gastric cancer is an important cause of cancer mortality and morbidity, and even with the current perioperative treatment, the relapse rate is still around 50%. New strategies for the perioperative setting are imperative, and new research in the area of targeted therapies and immunotherapy may improve the prognosis for each specific gastric cancer subpopulation. The perioperative treatment of gastric cancer is a fast-changing field, and the search for new biomarkers to guide treatment options and future trial designs is ongoing. ICIs may be incorporated in the perioperative setting for MSI-H and PD-L1 CPS-positive tumors, and results of the ongoing trials are awaited to guide patient selection. Additionally, other targets, such as TMB-H and HER-2, are under investigation with promising results, and novel biomarkers such as claudin 18.2 and FGFR-2 are just around the corner. In conclusion, all of this progress in perioperative strategies will allow for a more individualized, biomarker-driven treatment, using targeted therapies, immunotherapy, and the combination of multiple treatment modalities.

## Figures and Tables

**Table 1:** Currently approved perioperative gastric cancer therapies.

Clinical Trial (Phase)	n	Treatment	Tumor Type	Primary Endpoint	Outcomes	Reference
MAGIC (III)	503	ECF x3 → surg. → ECFx3 vs. surgery only	ES/EGJ/GC adenocarcinoma	OS	OS: HR 0.75; 95 CI 0.60 to 0.93; *p* 0.009 PFS: HR 0.66; 95 CI 0.53 to 0.81; *p* < 0.001	[11]
FNLCC ACCORD 07/FFCD 9703 (III)	224	FPx2-3 → surg. → FPx3-4 vs. surgery only	ES/EGJ/GC adenocarcinoma	OS	OS: HR 0.69; 95 CI 0.50 to 0.95; *p* 0.02 DFS: HR 0.65; 95 CI, 0.48 to 0.89; *p* 0.003	[12]
FLOT4-AIO(III)	716	FLOTx4 → surg. → FLOTx4ECF/ECXx3 → surg. → ECF/ECXx3	EGJ/GC adenocarcinoma ≥cT2 and/or cN+	OS	mOS: 50 mo vs. 35 mo; HR 0.77 95 CI 0.63 to 0.94]; *p* 0.012 mPFS, 30 mo vs. 18 mo; HR 0.75 95 CI 0.62 to 0.9; *p* 0.004	[10]

ECF: epirubicin, cisplatin, and 5-fluorouracil; ECX: epirubicin, cisplatin, and capecitabine; EGJ: esophagogastric junction; ES: esophagus; FLOT: 5-fluorouracil, leucovorin, oxaliplatin, and docetaxel; FP: 5-fluorouracil and cisplatin; GC: gastric cancer; HR: hazard ratio; OS: overall survival; PFS: progression-free survival; mOS: median overall survival; mPFS: median progression-free survival; n: number of patients randomized; surg.: surgery.

**Table 3:** Summary of phase II clinical trials exploring strategies with ICIs in the perioperative setting for locally advanced esophagogastric adenocarcinoma.

Clinical Trial (Phase)	n	Treatment	Primary Endpoint	Outcomes	Reference
GASPAR (II)	68	FLOTx4 + spartalizumabx2 → surg. → FLOTx4 + spartalizumabx2	pCR	pCR 31% TRG 1b 19% MPR 50%.	[22]
MONEO (II)	40	FLOT + Avelumabx4 → surg. → FLOT + Avelumabx4 → Avelumab 1y	pCR	Active, not recruiting	[23]
ICONIC (II)	34	FLOT + Avelumabx4 → surg. → FLOT + Avelumabx4	pCR	Closed early (unmet endpoint of pCR of 25% in 40 pts)	[24]
NEOSUMMIT01 (II)	108	Toripalimab + SOX/XELOXx3 → surg. → toripalimab + SOX/XELOXx3 → toripalimab 6 mo vs. SOX/XELOXx3 → surg. → SOX/XELOXx5	pCR, near-pCR	pCR (22.2% (12 of 54, 95 CI: 12.0–35.6%) vs. 7.4% (4 of 54, 95% CI: 2.1–17.9%); *p* = 0.030)	[25]
NCT0291816 (II)	36	XELOX + PEMBROx3 + PEMBROx1 → surg. → XELOX + PEMBROx3 → PEMBRO 1y	pCR	pCR 24.1%, near-pCR 17.6%	[26]
IMAGINE (II)	44	R: NIVOx6 → surg. → NIVOx4 → NIVO 1y NR: NIVOx2 → FLOT + NIVOx4 → surg. → FLOT + NIVOx4 R: NIVO + relatlimabx6 → surg. → NIVO + relatlimabx4 → NIVO 1y NR: NIVO + relatlimabx2 + NIVOx4 → surg. → NIVOx4 → NIVO 1y	pCR	Active, not recruiting	[27]

FLOT: 5-fluorouracil, leucovorin, oxaliplatin, and docetaxel; MPR: major pathologic response; n: number of patients randomized; near-pCR: near pathologic complete response; NIVO: nivolumab; NR: non-responders stratified by Early Response Evaluation according to trial design; pCR: pathological complete response; PEMBRO: pembrolizumab; R: responders stratified by Early Response Evaluation according to trial design; surg.: surgery; TRG: tumor regression grade; XELOX: capecitabine and oxaliplatin.

**Table 6:** Summary of clinical trials integrating VEGF-targeted agents in the perioperative treatment of gastric cancer with published results.

Clinical Trial (Phase)	VEGF Targeted Agent	n	Treatment	Outcomes	Status	Reference
ST03 (III)	Bevacizumab	1063	ECX + BEVAx3 → surg. → ECX + BEVAx3 vs. ECXx3 → surg. → ECXx3	OS 3y 48.1%	Published (negative)	[83]
RAMSES/FLOT7 (II/III)	Ramucirumab	152	FLOT + RAMx4 → surg. → FLOT + RAMx4 → RAMx16 vs. FLOTx4 → surg. → FLOTx4	pCR 26% R0 resection rate 96% vs. 82% (*p* 0.0093) mDFS 32 mo mOS 46 mo	Published (negative)	[85]
DRAGON-IV (II/III)	Apatinib	360	SOX + CAMx3 + APA x3 → surg. → SOX + CAMx3 + APA x3 → CAM + APA 1y vs. SOXx3 + APA x3 → surg. → SOXx3 + APA x3 → APA 1y vs. SOXx3 → surg. → SOXx3	MPR 51.1% (SOX + C+A) vs. 37.8% (SOX) pCR 18.3% (SOX + C+A) vs. 5.0% (SOX) (*p* < 0.0001)	Active, not recruiting	[86]
EPOC2001 (II)	Lenvatinib	32	Perioperative FLOT x8 + PEMBRO x8 ± LENVA daily → PEMBRO x11 ± LENVA daily for 1y	MPR 47% pCR 22%	Recruiting	[88]

APA: apatinib; BEVA: bevacizumab; CAM: camrelizumab; DFS: disease-free survival; ECX: epirubicin, cisplatin, and capecitabine; FLOT: 5-fluorouracil, leucovorin, oxaliplatin, and docetaxel; GC: gastric cancer; GEC: gastroesophageal cancer; LENVA: lenvatinib; MPR: major pathological response rate; OS: overall survival; PEMBRO: pembrolizumab; pCR: pathological complete response rate; RAM: ramucirumab; SOX: S-1 and oxaliplatin; surg.: surgery.
